# Antioxidant and Anti-Inflammatory Potential of Thymoquinone and Lycopene Mitigate the Chlorpyrifos-Induced Toxic Neuropathy

**DOI:** 10.3390/ph14090940

**Published:** 2021-09-20

**Authors:** Mohamed Aboubakr, Said M. Elshafae, Ehab Y. Abdelhiee, Sabreen E. Fadl, Ahmed Soliman, Afaf Abdelkader, Mohamed M. Abdel-Daim, Khaled A. Bayoumi, Roua S. Baty, Enas Elgendy, Amira Elalfy, Bodour Baioumy, Samah F. Ibrahim, Ahmed Abdeen

**Affiliations:** 1 Department of Pharmacology, Faculty of Veterinary Medicine, Benha University, Toukh 13736, Egypt; mohamed.aboubakr@fvtm.bu.edu.eg; 2 Department of Pathology, Faculty of Veterinary Medicine, Benha University, Toukh 13736, Egypt; said.alshafey@fvtm.bu.edu.eg; 3 Forensic Medicine and Toxicology Department, Faculty of Veterinary Medicine, Matrouh University, Matrouh 51744, Egypt; ehabyahya76@mau.edu.eg; 4 Biochemistry Department, Faculty of Veterinary Medicine, Matrouh University, Matrouh 51744, Egypt; nourmallak@mau.edu.eg; 5 Pharmacology Department, Faculty of Veterinary Medicine, Cairo University, Giza 12211, Egypt; galalpharma@cu.edu.eg; 6 Department of Forensic Medicine and Clinical Toxicology, Faculty of Medicine, Benha University, Benha 13518, Egypt; afaf.abdelkader@fmed.bu.edu.eg; 7 Department of Pharmaceutical Sciences, Pharmacy Program, Batterjee Medical College, Jeddah 21442, Saudi Arabia; abdeldaim.m@vet.suez.edu.eg; 8 Pharmacology Department, Faculty of Veterinary Medicine, Suez Canal University, Ismailia 41522, Egypt; 9 Department of Pathology, Faculty of Medicine, King Abdulaziz University, Jeddah 21442, Saudi Arabia; kabadr@kau.edu.sa; 10 Department of Forensic Medicine and Clinical Toxicology, Faculty of Medicine, Cairo University, Cairo 11956, Egypt; 11 Department of Biotechnology, College of Science, Taif University, P.O. Box 11099, Taif 21944, Saudi Arabia; rsbaty@tu.edu.sa; 12 Histology and Cell Biology Department, Faculty of Medicine, Benha University, Benha 13518, Egypt; enas.elgendy@fmed.bu.edu.eg (E.E.); amira.alalfay@fmed.bu.edu.eg (A.E.); 13 Department of Anatomy and Embryology, Faculty of Medicine, Benha University, Benha 13518, Egypt; bedor.bayuomi@fmed.bu.edu.eg; 14 Clinical Sciences Department, College of Medicine, Princess Nourah bint Abdulrahman University, Riyadh 11671, Saudi Arabia; 15 Department of Forensic Medicine and Toxicology, Faculty of Veterinary Medicine, Benha University, Toukh 13736, Egypt; 16 Center of Excellence for Screening of Environmental Contaminants (CESEC), Benha University, Toukh 13736, Egypt

**Keywords:** thymoquinone, lycopene, organophosphates, oxidative stress, inflammatory cytokines, caspase 3, neurotoxicity

## Abstract

CPF (chlorpyrifos) is an organophosphate pesticide used in agricultural and veterinary applications. Our experiment aimed to explore the effects of thymoquinone (TQ) and/or lycopene (LP) against CPF-induced neurotoxicity. Wistar rats were categorized into seven groups: first group served as a control (corn oil only); second group, TQ (10 mg/kg); third group, LP (10 mg/kg); fourth group, CPF (10 mg/kg) and deemed as CPF toxic control; fifth group, TQ + CPF; sixth group, (LP + CPF); and seventh group, (TQ + LP + CPF). CPF intoxication inhibited acetylcholinesterase (AchE), decreased glutathione (GSH) content, and increased levels of malondialdehyde (MDA), an oxidative stress biomarker. Furthermore, CPF impaired the activity of antioxidant enzymes including superoxide dismutase (SOD) and catalase (CAT) along with enhancement of the level of inflammatory mediators such as tumor necrosis factor-α (TNF-α), interleukin (IL)-6, and IL-1β. CPF evoked apoptosis in brain tissue. TQ or LP treatment of CPF-intoxicated rats greatly improved AchE activity, oxidative state, inflammatory responses, and cell death. Co-administration of TQ and LP showed better restoration than their sole treatment. In conclusion, TQ or LP supplementation may alleviate CPF-induced neuronal injury, most likely due to TQ or LPs’ antioxidant, anti-inflammatory, and anti-apoptotic effects.

## 1. Introduction

Chlorpyrifos (CPF), (*O*,*O*-diethyl-*O*-(3,5,6-trichloro-2-pyridyl) phosphorothioate), is part of a wide range of chlorinated organophosphate insecticides that is ubiquitously applied around the world to combat agricultural and domestic insects [1,2,3]. Alarmingly, CPF residues can persist for extended periods on the surfaces of water, plants, cereals, and fruits, posing sources of threat for humans and animals, mainly through dermal absorption, inhalation, or consuming contaminated food and drinking water [1]. The indiscriminate utilization of CPF has procured in mounting disquiet about their potential toxic impacts [4]. CPF and its metabolite chlorpyrifos oxon have the ability to prompt a variety of damaging effects on different body organs [1,3,5]. Due to the lipophilicity of CPF, the nervous system is a primary target for CPF; hence, it can facilely pass the blood–brain barrier and dismantle its stability, resulting in disruption of neuronal transmission and development of neurological disorders [5,6].

CPF has been reported to interfere with acetylcholinesterase (AchE) in central and peripheral nervous systems, allowing acetylcholine to accumulate in the synaptic cleft, resulting in uncontrolled cholinergic pathway activation and interrupting neuronal transmission [1,7]. Moreover, a growing body of research proposes that massive creation of damaging reactive oxygen species (ROS) is another possible mechanism implicated in CPF-induced neurotoxicity [8,9,10]. Oxidative stress is known to cause potential injuries to the cellular biomolecules including lipids, membranes, proteins, and DNA, leading to mitochondrial perturbation and ultimately apoptosis [1,3,9]. CPF has also been shown to enhance inflammatory responses by upregulating proinflammatory cytokines, especially tumor necrosis factor (TNF-α) and interleukin-1 (IL-1β) [9].

Natural antioxidants have recently gained worldwide attention due to their tremendous pharmacological potential and are now commonly used as alternative medicine. Among these, thymoquinone (TQ) is the principal bioactive ingredient derived from the volatile oil of *Nigella sativa* black seeds [11]. TQ has varied pharmacological benefits, including antioxidant and anti-inflammatory properties, by which TQ exerts its neuroprotective potential as well as treatment of many other diseases [8,11]. TQ antioxidant activity is ascribed to its potent capability to scavenge various ROS promoting the oxidant scavenging system by maintaining endogenous antioxidant enzyme property [12] and inhibiting lipid peroxidation [13]. Furthermore, TQ can reinstate the abnormal matrix metalloproteinase, lowering ROS levels [14]. TQ has also been proven to suppress proinflammatory mediators in various models based on inflammation, such as encephalitis, colitis, peritonitis, and arthritis [13]. Accordingly, a mounting body of literature reported that TQ has a neuroprotective potential against a variety of environmental chemicals such as malathion [15], microcystin [16], lead [17], and acrylamide [18].

Lycopene (LP) is an acyclic non-provitamin A belonging to the carotenoid family. It is abundantly found in red fruits and vegetables, including tomatoes, watermelon, pink grapefruit, beets, and pomegranate [19,20]. The potential effect of LP is mostly owing to its antioxidant [21], anti-inflammatory [22], and anti-apoptotic [23] effects. Because LP is extremely lipophilic and can easily penetrate the blood–brain barrier, it is plausible that it creates neuroprotective activity [19]. LP remediation has been found to improve oxidative stress-mediated neurologic lesions caused by methylmercury [24], aluminum [25], bisphenol A [26], and acrylamide [19]. The antioxidant power of LP is ascribed to the presence of conjugated double bonds with its efficacy to quench ROS. Since LP is not synthesized inside the body and its bioavailability is reduced with age and certain medical conditions, it is recommended to be supplemented daily [27].

There is a substantial evidence that TQ and LP can cross the blood–brain barrier and exert neuromodulatory effects [27,28,29]. Ahmad et al. have measured the concentration of TQ in brain homogenate using a UHPLC in an attempt to enhance the bioavailability of TQ to treat epilepsy in a rat model [29]. In another relevant study, LP could cross the blood–brain barrier and inhibit the aluminum-induced oxidative damage, inflammation, and apoptosis in rat hippocampal tissue [25]. Moreover, both TQ and LP are reported as safe chemicals [30,31,32]. Consistent with this assertion, we hypothesized that supplementing with TQ and/or LP could reduce CPF-prompted oxidative stress, inflammation, and promote brain tissue regeneration. Therefore, our research aimed to understand how effective TQ and/or LP supplementation were at reducing the chronic neurotoxic effects of CPF. Serum AchE activity, inflammatory cytokines, antioxidant activity, histopathological modulation, and caspase 3 expression were assessed in the brain.

## 2. Results

### 2.1. AchE Activity Evaluation

**Figure 1:**
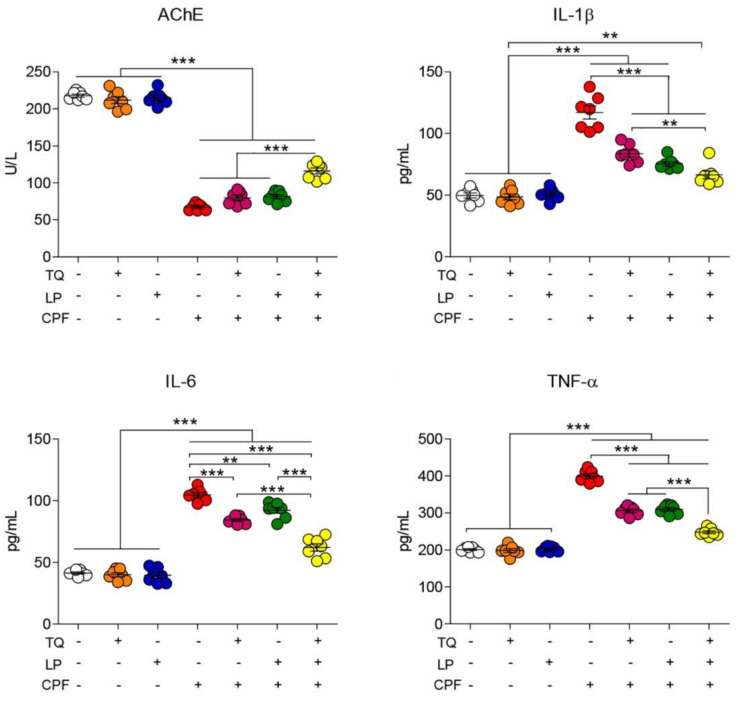
Dot plot of AchE activity and inflammatory cytokines after treatment with CPF, TQ, and LP. AchE, acetylcholinesterase; CPF, chlorpyrifos; IL-6, interleukin-6; IL-1β, interleukin-1β; LP, lycopene; TNF-α, tumor necrosis factor-α; TQ, thymoquinone. Values proffered as the mean ± SE (*n* = 7). ** *p* ≤ 0.01 and *** *p* ≤ 0.001.

As explicated in Figure 1, there were no significant changes in AchE activity in TQ and LP treated groups compared to controls. However, CPF intoxication provoked severe neurotoxicity presented by an outstanding decrease of AchE activity in serum. On the contrary, preconditioning of TQ or LP to rats (1 h prior to CPF exposure) led to a partial decrease of AchE activity. There was a noteworthy increase of AchE activity if TQ and LP were co-administrated together with CPF when matched to their sole treatment. These observations suggest that TQ and/or LP treatment modulate CPF-induced neurological injuries.

### 2.2. Serum Proinflammatory Cytokines Assessment

As depicted in Figure 1, neurotoxicity and brain inflammation were induced after CPF exposure, elucidated by a substantial (*p* ≤ 0.05) increase in TNF-α, IL-1β, and IL-6 levels in rat serum when compared to controls. Contrariwise, a decreased toxic impact of CPF was spotted when CPF-intoxicated rats were treated by TQ or LP, indicated by amendment of all proinflammatory cytokines levels. Combined CPF treatment with both remedies (TQ and LP) could evidently labor more worthy betterment of those parameters. These findings confirm that the amelioration of CPF-induced damage that exerted subsequent to TQ or/and LP administration was due to their anti-inflammatory effect. Expectedly, our data revealed the safety of TQ and LP, indicated by no alterations, were observed in the measured proinflammatory cytokines.

### 2.3. Brain Lipid Peroxidation and Antioxidant Indices

**Figure 2:**
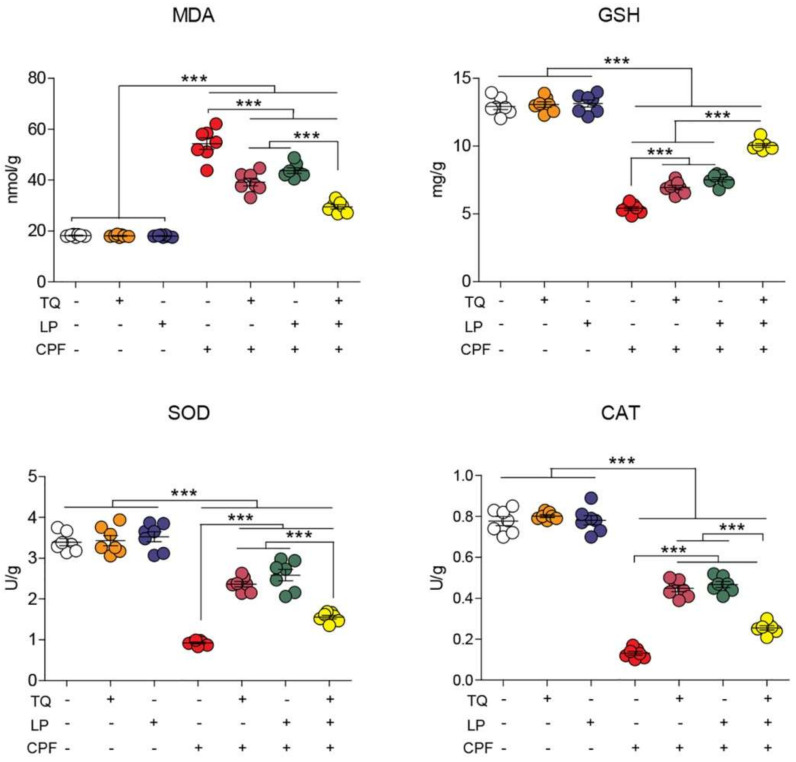
Dot plot of oxidative/antioxidative status after treatment with CPF, TQ, and LP. CAT, catalase; CPF, chlorpyrifos; GSH, reduced glutathione; LP, lycopene; MDA, malondialdehyde; SOD, superoxide dismutase; TQ, thymoquinone. Values are proffered as the mean ± SE (*n* = 7). *** *p* ≤ 0.001.

Lipid peroxidation marker (MDA) and antioxidant enzyme activity (CAT, SOD, and GSH) following CPF, TQ, or LP administration are displayed in Figure 2. As depicted, TQ and LP groups did not show any negative impact (*p* > 0.05) on oxidative stress markers. However, CPF exposure prompted marked oxidative stress indicated by drastic increases in the MDA levels alongside an outstanding decrease in CAT and SOD activity and GSH concentration in brain tissues with respect to the control group (*p* ≤ 0.05). TQ or LP treatment notably attenuated the brain oxidative harm inflicted by CPF-intoxication. More remarkable improvement of oxidative state in group VII (TQ + LP + CPF) in confronting group V and VI suggests that concurrent use of TQ and LP has potent synergistic antioxidant properties against CPF toxicity.

### 2.4. Histopathological Alteration

**Figure 3:**
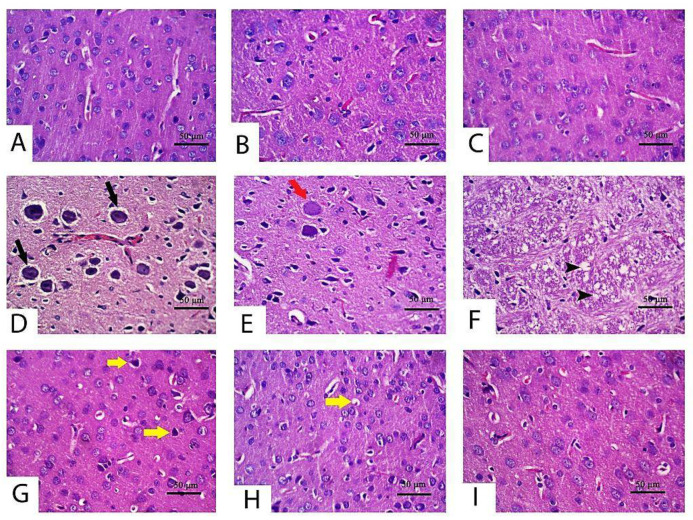
Histopathology of the cerebrum in control and CPF, TQ, and LP treated groups (H&E stain). Apparently, normal neurons were observed in control (**A**), TQ (**B**) and LP (**C**) treated rats with null to minimal apoptotic neurons. (**D**–**F**) Cerebral cortex of CPF-treated rats showing neuronal degeneration (tigrolysis; black arrow) (**D**), central chromatolysis (red arrow) (**E**) and neuropile vacuolation (black arrowhead) (**F**). A low number of shrunken apoptotic neurons (yellow arrows) was observed in TQ (**G**), LP (**H**) and combination (**I**) groups co-treated with CPF.

The histopathological changes were assessed in the cerebrum and cerebellum tissue following CPF exposure to emphasize the obtained findings. As considered to cerebral cortex tissue specimen (Figure 3; control (Figure 3A), TQ (Figure 3B) and LP (Figure 3C) groups) displayed normal features of histological architecture of the cerebral cortex. Contrariwise, the cerebral cortex following CPF intoxication exhibited severely degenerated to necrotic neurons. Degenerated neurons had intracytoplasmic vacuoles, vague cell boundaries, with a significant number of degraded cell residue structures associated with inflammatory cell infiltrations, while necrotic neurons were characterized by pyknotic nuclei with the presence of satellitosis (Figure 3D). Certain necrotic neurons showed tigrolysis with central chromatolysis (Figure 3E). In addition, CPF induced severe vacuolation in the neuropil (Figure 3F). Focal areas of malacia were observed in the cerebral cortex. Severe congestion and hemorrhage of blood vessels were also pronounced in this group. With concurrent use of CPF and TQ, there was a marked reduction in the number of degenerated and necrotic neurons compared to untreated CPF rats (Figure 3G). Moreover, there was mild congestion of blood capillaries with no evidence of vacuolation in the neuropil. Foci of degenerated/necrotic neurons, gliosis, and neuronophagia were still observed in the cerebral cortex in CPF + LP treated rats (Figure 3H). The neuroprotective effect was more distinct in the CPF + TQ + LP treated group, expounded by a great improvement in the histopathological lesions induced by CPF. Congestion of meningeal blood vessels and the presence of few shrunken neurons were the only findings in the cerebral cortex in these groups (Figure 3I).

**Figure 4:**
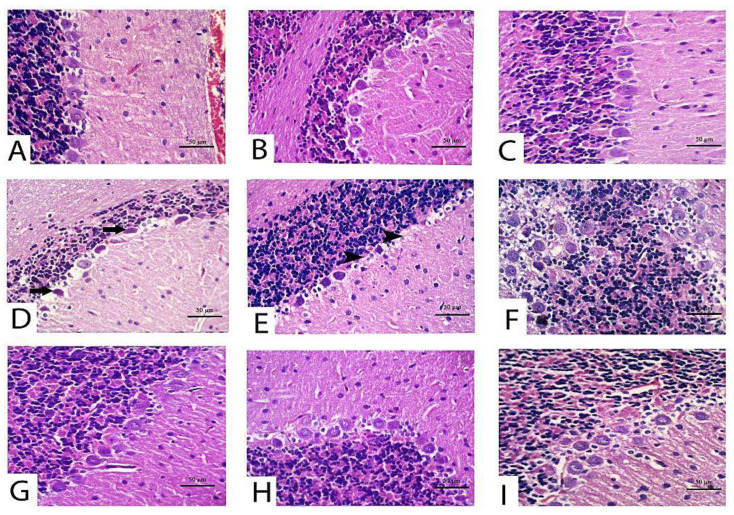
Histopathology of the cerebellum in control and CPF, TQ, and LP treated groups (H&E stain). Nearly normal Purkinje neurons were observed in control (**A**), TQ- (**B**) and LP- (**C**) treated rats with null to mild degeneration of few neurons. (**D**–**F**) Cerebellum of CPF treated rats showing Purkinje cell apoptosis (black arrows) (**D**), loss (black arrowheads) (**E**) and disorganization with molecular cell layer (**F**). Marked decreases in degenerated/apoptotic Purkinje cells were observed in TQ (**G**), LP (**H**) and combination (**I**) groups co-treated with CPF.

With respect to cerebellum histological screening of control, TQ and LP-treated groups revealed normal architecture of brain tissues as exhibited in Figure 4A (control), Figure 4B (TQ), Figure 4C (LP). Contrariwise, CPF-intoxicated rats evinced that most Purkinje cells were shrunken with karyolitic nuclei and variable degrees of chromatolysis (Figure 4D). Loss in Purkinje cells, satellitosis, and neuronophagia were the predominant findings in most sections in this group (Figure 4E). Disorganization of the molecular cell layer was also observed between the Purkinje cell layer and other layers in certain areas (Figure 4F). Additionally, there were impoverished dendrites in certain areas of the cerebellar cortex. On the contrary, with the concurrent use of TQ or LP with CPF intoxication, the cerebellar architecture was relatively restored to a normal picture in comparison to untreated CPF rats (Figure 4G, CPF + TQ and Figure 4H, CPF + LP). There was a significant decrease in the severity and distribution of cerebellar lesions in these groups. A decrease in the number of necrotic Purkinje cell layer in the cerebellar cortex and restoration of molecular cell layer was observed. In CPF + TQ + LP treated rats, remarkable amelioration of pathological alterations was noticed (Figure 4I).

Along with the forementioned biochemical data, there were no histological alterations detected either in the cerebrum or cerebellum after treatment with TQ or LP.

### 2.5. Immunohistochemical Data

**Figure 5:**
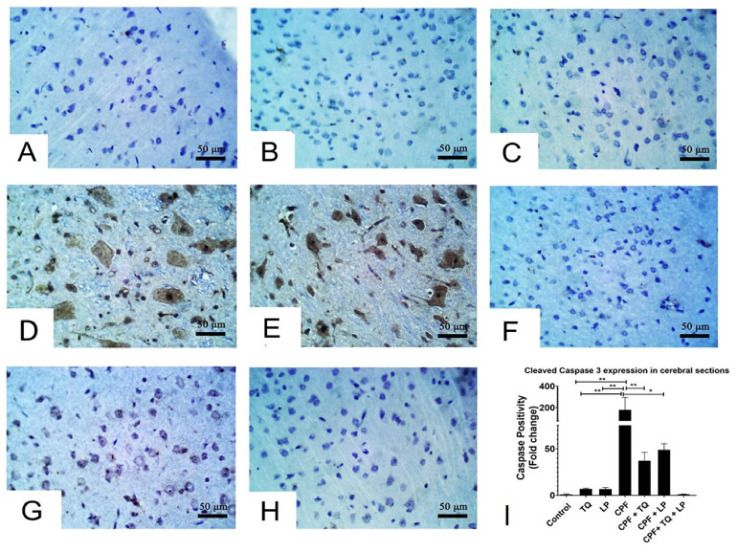
Immunohistochemical staining of cleaved caspase 3 in cerebral sections. (**A**–**C**) Almost all the neurons in control (**A**), TQ- (**B**) and LP- (**C**) treated groups had no cleaved caspase 3 staining. (**D**,**E**) Many neurons displayed strong staining for cleaved caspase 3 in CPF intoxicated rats. (**F**–**H**) Few neurons were weakly to moderately positive for cleaved caspase 3 in CPF intoxicated rats co-treated with TQ (**F**), LP (**G**) or both (**H**). (**I**) Bar graph represents the relative cleaved caspase 3 positivity in different groups in contrast to CPF intoxicated group. Substantial differences are exhibited as: * *p* ≤ 0.05, ** *p* ≤ 0.01.

Alterations in cleaved caspase 3 expressions following treatment with CPF, TQ and/or LP in cerebral sections are presented in Figure 5. CPF intoxication distinctly boosted cleaved caspase 3 expressions in the cytoplasm and nuclei of neurons and glial cells in the cerebral sections elucidated inception of the apoptotic pathways, contrary to control group (Figure 5D,E). Co-administration of TQ (Figure 5F) or LP (Figure 5G) or both (Figure 5H) could mitigate the CPF-prompted damage displayed by a decline in cleaved caspase 3 protein expression. The reduction was more pronounced in TQ (Figure 5F), and TQ + LP (Figure 5H) treated groups versus LP- (Figure 5G) treated groups after CPF intoxication. Despite the fact that LP + CPF treated rats had less cleaved caspase 3 activity than CPF intoxicated group, few neurons showed weak to moderate staining of cleaved caspase 3 (Figure 5H). Cleaved caspase 3 positivity (positive cells/total cells) was quantified in all groups and the data were analyzed statistically utilizing one-way ANOVA. Compared to CPF intoxicated group, the positivity of cleaved caspase 3 was significantly reduced in control (untreated and TQ or LP treated) groups and CPF rats cotreated with TQ, LP, or TQ + LP combination (Figure 5I).

**Figure 6:**
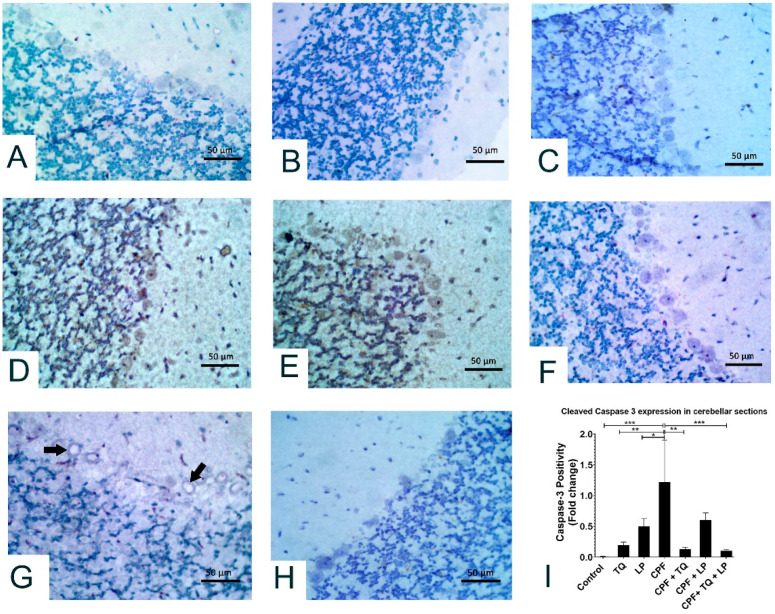
Immunohistochemical staining of cleaved caspase 3 in the cerebellum. All the neurons in molecular, Purkinje and granular cell layers displayed no caspase 3 staining in control (**A**), TQ- (**B**) and LP- (**C**) treated rats. (**D**,**E**) Most Purkinje cells and many neurons in granular and molecular cell layers showed strong caspase 3 staining in CPF intoxicated rats. (**F**) Most neurons in all the cerebellar layers had no caspase 3 staining in TQ + CPF treated rats. (**G**) Certain Purkinje cells were vacuolated and moderately stained with cleaved caspase 3 in LP + CPF treated rats. (**H**) All the neurons in the cerebellar sections were devoid of caspase 3 staining in TQ + LP + CPF treated rats. (**I**) Bar graph represents the relative cleaved caspase 3 positivity in cerebellar sections of different groups compared to CPF intoxicated group. Substantial differences are exhibited as: * *p* ≤ 0.05, ** *p* ≤ 0.01, and *** *p* ≤ 0.001.

Alterations in cleaved caspase 3 expressions after treatment with CPF, TQ, and/or LP in cerebellar sections are presented in Figure 6. In control, TQ and LP treated rats, all the neurons in molecular, Purkinje and granular cell layers had no detectable level of cleaved caspase 3 protein expression (Figure 6A–C). CPF intoxication promoted cleaved caspase 3 expression in the cytoplasm and nuclei of numerous neurons in the Purkinje cell layer and few neurons in the granular and molecular cell layers, and certain glial cells were also positive to caspase 3 in the molecular cell layer, demonstrating inception of the apoptotic pathways in contrast to control group (Figure 6D,E). Reduction in cleaved caspase-positive Purkinje cells was observed in CPF-intoxicated rats if co-treated with TQ, LP, or combined (Figure 6F–H). Cleaved caspase 3 staining was distinctly reduced if CPF was concurrently used with TQ or TQ + LP compared to LP treated group (Figure 6G). Cleaved caspase 3 positivity (positive cells/total cells) was quantified in all groups and the data were analyzed statistically utilizing one-way ANOVA. Compared to CPF intoxicated rats, the proportion of cleaved caspase 3 positive cells was dramatically lowered in control, TQ, and LP groups as well as in the CPF rats co-treated with TQ or TQ + LP. There was a tendency for cleaved caspase 3 to be decreased in LP + CPF group but was not statistically significant (Figure 6I). In addition, the caspase 3 expression exhibited normal levels of expression in TQ and LP groups when compared to control group.

## 3. Discussion

CPF is an organophosphorus pesticide that is profoundly used to combat a variety of insects. Environmental, occupational, and dietary exposure to CPF have all been implicated as health concerns in both animals and humans, with various deleterious outcomes [3,6,10]. The brain is deemed the primary target for CPF toxicity; in particular, its repeated exposure causes various extents of neuronal deterioration [33]. CPF functions as a neurotoxic by suppressing AchE, a membrane-bound enzyme essential for acetylcholine (Ach) metabolism at the neuro-muscular junctions and cholinergic synapses [34]. CPF inhibits AchE irreversibly by phosphorylating the enzyme’s serine OH group, leading to Ach buildup in the brain and hyperstimulation of the cholinergic nerve system, resulting in a variety of clinical disorders [35]. Along with the aforementioned, the current work affirmed that CPF induced neurotoxicity was elucidated by a substantial decrease in AchE levels in CPF-intoxicated rats compared to controls. AchE is a pivotal enzyme in the nervous system; wherefore, it is considered as a standard biomarker for organophosphate-induced neurotoxicity [36]. These findings are congruent with those obtained by preceding research by AlKahtane et al. [7], Fereidouni et al. [37], and Mahmoud et al. [10], who demonstrated that CPF induces neurotoxicity associated with lowered levels of AchE. Similarly, decreased serum levels of AchE may be a reverberation of its diminished activity in the brain after CPF-stimulated cholinergic transmission disruption [5].

In addition to AchE suppression, other evidence proposed that excessive production of ROS and lipid peroxidation (LPO) are other mechanisms implicated in CPF-induced neuropathy [5,9,10]. ROS (hydroxyl radical, OH^•^; hydrogen peroxide, H_2_O_2_; superoxide anion, O_2_^•−^) and reactive nitrogen species (nitric oxide, NO and peroxynitrite, ONOO^−^) are reported to be invigorated during CPF intoxication as a result of exhaustion of the endogenous antioxidant molecules [5,7,35]. Verma et al. [6] has documented alterations in the antioxidant system, including superoxide dismutase (SOD), catalase (CAT), and reduced glutathione (GSH) in CPF-intoxicated rats. Thence, oxidative damage of the brain is triggered by several mechanisms such as enhancement of LPO, mitochondrial disruption, DNA damage, protein unfolding, and changes in pH and the intracellular calcium brought about the promotion of cell death [7]. Consistently, the current study expounds that ROS contributed to the CPF-induced neurotoxicity, as indicated by a dramatic reduction in the enzymatic activities of SOD and CAT, as well as decreases in GSH level detected in the brain tissue. It is well known that GSH is an essential endogenous non-enzymatic antioxidant that boosts free radical detoxification. The cysteine residue of GSH provides a nucleophilic thiol that is paramount in the detoxification of electrophilic metabolites. Besides, GSH is important for the regeneration of the enzymatic antioxidants, GPx and glutathione-S-transferase (GST), and is required for maintaining cellular antioxidant competence [35,38].

Moreover, SOD is an endogenous enzyme that functions as the initial line of enzymatic defense in mitochondria, rapidly catalyzing dismutase O_2_^•−^ to O_2_ and H_2_O_2_; thereafter, CAT degrades the formed H_2_O_2_ to water in a process that helps to quench the induced oxidative damage [39]. However, in a condition where CAT is exhausted, Fenton’s reaction occurs and produces large amounts of OH^•^ (the most harmful reactive radical), which aggressively attacks the membrane lipids, triggering LPO and formation of MDA [40]. MDA, a well-known marker for LPO, indicates cell injury incurred by phospholipid breakdown. MDA can also interact with other vital subcellular molecules such as DNA and proteins, which complicates the matters. The present investigation revealed worthy increases in MDA levels, which affirms the existence of membrane injury. In consonance with these findings are those of Aly et al. [38], who confirmed enhanced MDA production after CPF exposure. Our oxidative/antioxidants appeared consistent with those obtained by previous reports that revealed incompetency in the cellular antioxidants in the neuronal tissue following CPF exposure [9,10]. Besides, Albasher et al. [5] has used the CPF as a model for induction of oxidative hurt in the brain tissue. Because of the higher lipid content of the brain, lower antioxidant levels, and rapid oxygen exhaustion, the brain was more vulnerable to the progression of oxidative stress compared to other body organs. The high lipophilicity of CPF property plays a central role in potentiating the LPO in brain tissue [6]. These results were confirmed by the histopathological screening, which emphasizes the presence of LPO in the neuronal cell membrane, as indicated by brain cell degeneration, vague cell boundaries, thin cytoplasm, and darkly spotted nuclei with a significant number of degraded cell remnant structures.

Another finding has been spotted in the current research, remarkable enhanced levels of proinflammatory cytokines; IL-1β, IL-6, and TNF-α, following CPF intoxication. CPF exposure causes induction of acute-phase inflammatory reactions, resulting in generation of such cytokines, supposing another mechanism involved in CPF-induced neuronal injuries [10]. Oxidative stress and enhanced generation of ROS are thought to trigger an intracellular signaling cascade that increases the expression of proinflammatory genes and the release of inflammatory cytokines, leading to a heavy inflammatory response [7]. Our histopathological examination assured this impact, which was indicated by remarkable inflammatory cell infiltrations in brain tissues. Our findings confirm those obtained by Albasher et al. [5], AlKahtane et al. [7], and Mahmoud et al. [10].

Increasing evidence suggests that CPF promotes apoptosis via producing ROS, which alters mitochondrial membrane potency, resulting in the liberation of the cytochrome c into the cytosol, triggering the cleaved caspase 3; thereby, the apoptotic cascade is initiated [41]. In consistence, the present study showed cellular apoptosis observed by an upregulated expression of activated caspase 3 protein after CPF-intoxication in the cortical tissue.

TQ is reported to have antioxidant and anti-inflammatory activities executed by ROS scavenging power and antioxidant boosting properties [16]. LP is also substantive to have the same beneficial pharmacological activities. In the current study, treatment with TQ and/or LP weakened CPF-induced neurotoxicity as shown by noteworthy restoration of serum AchE levels, substantial reductions of proinflammatory cytokines, and amendment of the oxidative/antioxidative status. In addition, refinement of neuro-histological changes as well as reduction of apoptosis (indicated by downregulation of caspase 3 expression) was noticed in our study after TQ and LP supplementations. The physicochemical properties of TQ and LP are pivotal in their antioxidant potentiality. Their chemical structures comprise a phenol ring formed of carbon and hydrogen atoms (H^+^)(LP; C40H56, TQ;C10H12O2) with several double bonds conferring free delocalization of electrons and offering a generous source of H^+^ essential for neutralization of ROS; herein, more functional GSH becomes free in the cytosol, improving the antioxidant state [20]. Previous researches proposed various implied mechanisms for the antioxidant potential of TQ, which includes forthright interaction of TQ with GSH or NADP to form thymohydroquinone or glutathionyldihydro-TQ complexes that are able to quench ROS and boost the expression of antioxidant genes [42,43].

LP is a lipophilic natural agent that has the advantage of incorporating with the lipid bilayer in the cell membrane. Since ROS can directly interact with the cell membrane’s unsaturated fatty acids content, causing LPO, LP can act as a substrate for the harmful ROS instead. During this process, more H^+^ is abstracted from LP rather than unsaturated fatty acids, protecting the neuronal membrane from the impact of CPF-induced LPO [44,45], which is evidenced in this study by a substantial decrease in MDA levels. Furthermore, LP can attenuate oxidative stress by triggering the nuclear factor-2 erythroid related factor-2 pathway, enhancing the antioxidant genes and proteins [46]. Moreover, previous researches have shown that LP exerts its anti-inflammatory effect via suppressing the NF–κB pathway and limiting the generation of proinflammatory cytokines, such as TNF and ILs [47].

Along with the previous reports [30,31,32], the present work also affirms the extreme safety of both natural antioxidants (TQ and LP), indicated by no changes in biochemical parameters, histopathological data, and caspase 3 expression. LD_50_ of TQ was recorded at 794 and 2400 mg/kg in rats and mice, respectively, when given orally [48]. In another study conducted by Jonker and his group demonstrated that LP is safe at high doses: 586 mg/kg in male and 616 mg/kg body weight in female rats when administrated orally for 90 days [30].

**Figure 7:**
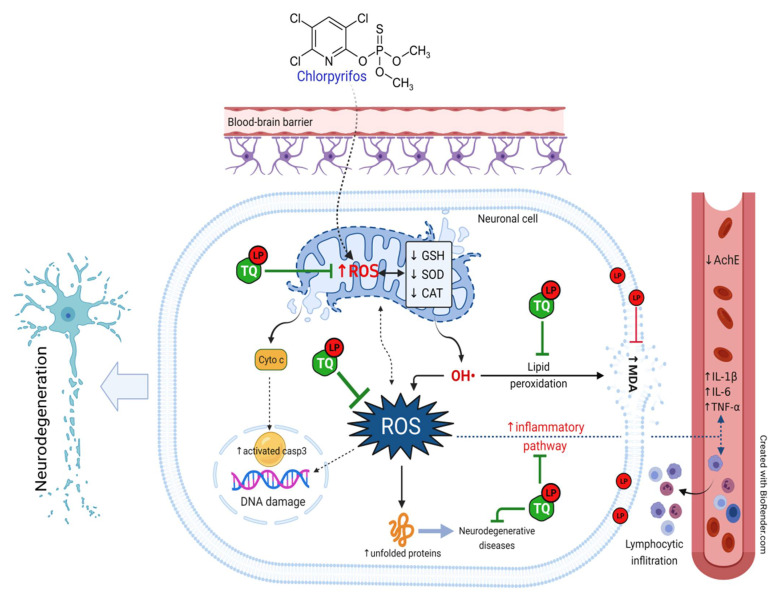
Schematic diagram summarizes the mechanistic insights behinds the protective potential of TQ and LP during CPF-induced neurotoxicity. AchE, acetylcholinesterase; CAT, catalase; CPF, chlorpyrifos; GSH, reduced glutathione; IL- 6, interleukin-6; IL-1β, interleukin-1β; LP, lycopene; MDA, malondialdehyde; ROS, reactive oxygen species; SOD, superoxide dismutase; TNF-α, tumor necrosis factor-α; TQ, thymoquinone.

In this study, co-supplementation of TQ and LP offered an abundance of phenol rings and H^+^ atoms by which they could distinctly amend all oxidative and inflammatory biomarkers in an excellent way versus their discrete treatments. These mechanisms were also reflected in the progression of cell apoptosis in the brain tissue exhibited by observable downregulation of the cleaved caspase 3 expression after CPF-intoxication. Taken all together, it is assumed that the promoted ameliorative effects of their simultaneous administration were attributable to mainly the synergistic antioxidant activity of both remedies. Figure 7 summarizes the mechanistic insights behind the protective potential of TQ and LP during CPF-induced neurotoxicity.

## 4. Materials and Methods

### 4.1. Chemicals

CPF was taken up from Egyptian Pesticides and Chemicals Company (EPIC), Cairo, Egypt. TQ and LP were supplied from Sigma Aldrich (Saint Louis, MO, USA), purity ≥ 98% and 90%, respectively. AchE was obtained from R&D (Mannheim, Germany). Interleukin-1β (IL-1β) and interleukin-6 (IL-6) were gained from Glory Science Co. Ltd. (Del Rio, TX, USA) and tumor necrosis factor-α (TNF-α) was bought from BioSource International Inc. (Camarillo, CA, USA). Diagnostic kits for assessment of malondialdehyde (MDA), reduced glutathione (GSH), catalase (CAT), and superoxide dismutase (SOD) were gained from Biodiagnostic Company (Cairo, Egypt).

### 4.2. Animals and Ethical Statement

Male Wistar Albino rats weighing 185–215 g were obtained from the Egyptian Organization for Biological Products and Vaccines. Rats were fed a conventional pellet diet and had free access to water under a controlled environment (25 °C temperature, 12:12 h light/dark cycle, and 45–55% humidity). All animal treatments and experimental procedures were conducted according to the directory of laboratory animals care and use and approved by the Faculty of Veterinary Medicine Ethical Committee of Research, Benha University, Egypt (approval no: BUFVTM 05-03-21).

### 4.3. Experimental Protocol

After acclimation, experimental rats were assigned into 7 equable groups (7 rats each). Group I (Control): rats received corn oil only as a vehicle. Group II (TQ): rats received TQ (10 mg/kg, orally via gavage) [15]. Group III (LP): rats were given LP (10 mg/kg, orally via gavage) [19]. Group IV (CPF): rats received CPF (10 mg/kg, orally via gavage) [10]. Group V (TQ + CPF): rats were given TQ and CPF. Group VI (LP + CPF): rats were given LP and CPF. Group VII (TQ + LP + CPF): rats were treated with TQ, LP, and CPF at the same doses mentioned above. Notably, TQ and LP were administrated one hour prior to CPF. All of the treatments were administered orally once a day for 28 sequential days.

### 4.4. Samples Collection and Processing

After 24 h had passed since the preceding treatment, entire groups were euthanized by xylazine: ketamine mixture (1:1) and 0.15 mL/100 gm body weight intraperitoneally. Blood specimen was gathered forthwith from retro-orbital venous plexus and centrifuged for 15 min at 1200× *g*. Serum was collected and preserved at −20 °C for analysis of AchE and proinflammatory mediators (IL-1β, IL-6, and TNF-α) levels. The brain was quickly harvested and suffused in cold physiological saline to get rid of blood clots and RBCs, and then wiped dry with a filter paper. Thereafter, each tissue sample was split into two portions; one portion was kept in a 10% buffered formalin for forthcoming histoarchitecture and immunohistochemically inspections. The other portion was processed as subsequently mentioned for oxidative stress biomarkers assessment.

### 4.5. Assessment of Serum AchE Activity and Proinflammatory Biomarkers

The activity of AchE was assessed according to the guidelines set out by Ellman et al. [46]. Proinflammatory cytokines (IL-1β, IL-6, and TNF-α) were measured utilizing commercialized ELISA reagents according to the manufacturer’s directives, and the absorbance estimates were assessed using an automated ELISA analyzer at 450 nm.

### 4.6. Assays of Oxidative Stress Parameters

One gram of tissue sample was homogenized in ice-cold buffered sol (K_3_PO_4_ 50 mmol, EDTA 1 mmol, pH 7.5) by means of an electrical homogenizer. Then, the resultant homogenate was centrifuged in a cooling centrifuge (5000 rpm till 10 min at 4 °C). The supernatant was aliquoted and kept at −80 °C for assessment of MDA levels and activities of GSH, SOD, and CAT, utilizing special kits from Laboratory Biodiagnostic Co., Giza, Egypt.

### 4.7. Histoarchitecture and Immunohistochemical Examination

After appropriate fixation (in buffered 10% formalin for a minimum of 24 h at room temperature), the harvested brain tissue specimens (cerebrum and cerebellum) were rinsed down the flood of faucet water for 10 min and thereafter dehydrated by immersion in sequent ethanol dilutions. Afterward, they cleared up in xylene solution. Specimens were incorporated into paraffin at 60 °C and severed (5 μm) before being stained with hematoxylin and eosin (H&E) to examine the histoarchitectural alterations by a bright-field microscope.

For immunohistochemical assessment, the paraffin blocks were dewaxed and desiccated by inundations in consecutive solutions of ethanol. Afterward, antigen retrieval was accomplished by processing tissue slices with antigen retrieval solution for 50 min in 10 mM citrate buffer (pH 6.0) using a steamer, followed by slow cooling. The peroxidases were repressed by utilizing a 3% H_2_O_2_ sol for 30 min and then washed in PBS 3 times (each for 5 min). Goat serum (5%) block up solution was then added for 20 min to block non-specific places (DAKO X 0907, Carpinteria, CA, USA) at room temperature for 2 h. Incubation with mouse anti-rat polyclonal anti-activated caspase 3 antibody (Neomarkers, Fremont, CA, USA; dilution 1:50) occurred overnight at 4 °C. Subsequently, the slide was washed out 3 times with PBS before being incubated using biotinylated anti-mouse IgG (DAKO LSAB 2 Kit) for 1 h at 37 °C. Ultimately, the brown staining was evident with 3,3-diaminobenzidine tetrahydrochloride (DAB; Dako, Tokyo, Japan) and the slide counterstained with Mayer’s hematoxylin. Image J software was used to record staining intensity and positivity.

### 4.8. Statistical Analysis

Statistical analyses and visualization were completed using GraphPad PRISM 7.0 (San Diego, CA, USA). The significant divergence through multiple groups comparisons were analyzed by one-way ANOVA and Duncan test as a post hoc test was used. Concerning immunohistochemistry scoring, the data from whole groups were normalized against the control group and graphed. Values are expressed as mean ± SE and considered statistically respectable at *p* ≤ 0.05.

## 5. Conclusions

CPF evoked noteworthy neurotoxic effects in the rat brain indicated by reduced serum AchE levels, increased proinflammatory cytokines, and alteration of the oxidative state. TQ and LP possess the power to conserve the neurons from CPF-prompted neurotoxicity, possibly by counteracting oxidative stress and apoptosis, which may be ascribed to their antioxidant and anti-inflammatory properties. Supplementation with TQ or LP only mitigates such damage in CPF-intoxicated animals. A combined treatment of both remedies exerts more betterment than their individual administration.

## Data Availability

Upon request, the data utilized to verify the findings of this research are obtainable from the corresponding authors.

## Abbreviations

AchE, acetylcholinesterase; CAT, catalase; CPF, chlorpyrifos; GSH, reduced glutathione; IL-6, interleukin-6; IL-1β, interleukin-1β; LP, lycopene; MDA, malondialdehyde; ROS, reactive oxygen species; SOD, superoxide dismutase; TNF-α, tumor necrosis factor-α; TQ, thymoquinone.
