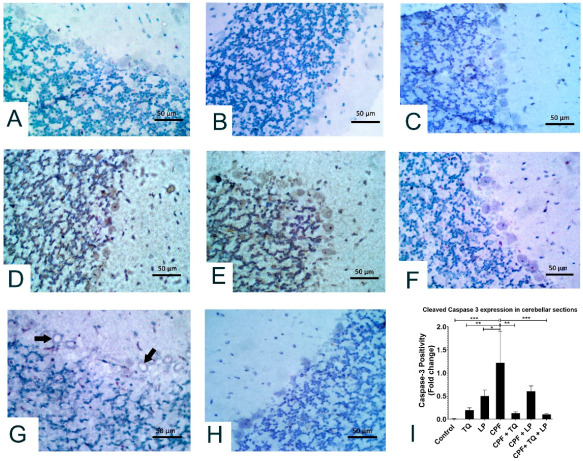# Correction: Aboubakr et al. Antioxidant and Anti-Inflammatory Potential of Thymoquinone and Lycopene Mitigate the Chlorpyrifos-Induced Toxic Neuropathy. *Pharmaceuticals* 2021, *14*, 940

**DOI:** 10.3390/ph18050745

**Published:** 2025-05-19

**Authors:** Mohamed Aboubakr, Said M. Elshafae, Ehab Y. Abdelhiee, Sabreen E. Fadl, Ahmed Soliman, Afaf Abdelkader, Mohamed M. Abdel-Daim, Khaled A. Bayoumi, Roua S. Baty, Enas Elgendy, Amira Elalfy, Bodour Baioumy, Samah F. Ibrahim, Ahmed Abdeen

**Affiliations:** 1 Department of Pharmacology, Faculty of Veterinary Medicine, Benha University, Toukh 13736, Egypt; mohamed.aboubakr@fvtm.bu.edu.eg; 2 Department of Pathology, Faculty of Veterinary Medicine, Benha University, Toukh 13736, Egypt; said.alshafey@fvtm.bu.edu.eg; 3 Forensic Medicine and Toxicology Department, Faculty of Veterinary Medicine, Matrouh University, Matrouh 51744, Egypt; ehabyahya76@mau.edu.eg; 4 Biochemistry Department, Faculty of Veterinary Medicine, Matrouh University, Matrouh 51744, Egypt; nourmallak@mau.edu.eg; 5 Pharmacology Department, Faculty of Veterinary Medicine, Cairo University, Giza 12211, Egypt; galalpharma@cu.edu.eg; 6 Department of Forensic Medicine and Clinical Toxicology, Faculty of Medicine, Benha University, Benha 13518, Egypt; afaf.abdelkader@fmed.bu.edu.eg; 7 Department of Pharmaceutical Sciences, Pharmacy Program, Batterjee Medical College, Jeddah 21442, Saudi Arabia; abdeldaim.m@vet.suez.edu.eg; 8 Pharmacology Department, Faculty of Veterinary Medicine, Suez Canal University, Ismailia 41522, Egypt; 9 Department of Pathology, Faculty of Medicine, King Abdulaziz University, Jeddah 21442, Saudi Arabia; kabadr@kau.edu.sa; 10 Department of Forensic Medicine and Clinical Toxicology, Faculty of Medicine, Cairo University, Cairo 11956, Egypt; 11 Department of Biotechnology, College of Science, Taif University, P.O. Box 11099, Taif 21944, Saudi Arabia; rsbaty@tu.edu.sa; 12 Histology and Cell Biology Department, Faculty of Medicine, Benha University, Benha 13518, Egypt; enas.elgendy@fmed.bu.edu.eg (E.E.); amira.alalfay@fmed.bu.edu.eg (A.E.); 13 Department of Anatomy and Embryology, Faculty of Medicine, Benha University, Benha 13518, Egypt; bedor.bayuomi@fmed.bu.edu.eg; 14 Clinical Sciences Department, College of Medicine, Princess Nourah bint Abdulrahman University, Riyadh 11671, Saudi Arabia; 15 Department of Forensic Medicine and Toxicology, Faculty of Veterinary Medicine, Benha University, Toukh 13736, Egypt; 16 Center of Excellence for Screening of Environmental Contaminants (CESEC), Benha University, Toukh 13736, Egypt

## Error in Figure 6H

In the original publication, there was a mistake in Figure 6H as published [1]. An uncorrected image was unintentionally inserted into the manuscript. The corrected Figure 6 appears below. The authors state that the scientific conclusions are unaffected. This correction was approved by the Academic Editor. The original publication has also been updated.